# Reversal of senescence by N resupply to N-starved *Arabidopsis thaliana*: transcriptomic and metabolomic consequences

**DOI:** 10.1093/jxb/eru119

**Published:** 2014-04-01

**Authors:** Salma Balazadeh, Jörg Schildhauer, Wagner L. Araújo, Sergi Munné-Bosch, Alisdair R. Fernie, Sebastian Proost, Klaus Humbeck, Bernd Mueller-Roeber

**Affiliations:** ^1^University of Potsdam, Institute of Biochemistry and Biology, Karl-Liebknecht-Straße 24–25, Haus 20, D-14476 Potsdam-Golm, Germany; ^2^Max-Planck Institute of Molecular Plant Physiology, Plant Signalling Group, Am Muehlenberg 1, D-14476 Potsdam-Golm, Germany; ^3^Martin-Luther-University Halle-Wittenberg, Institute of Biology, Weinbergweg 10, D-06120 Halle, Germany; ^4^Max-Planck Institute of Molecular Plant Physiology, Central Metabolism Group, Am Muehlenberg 1, D-14476 Potsdam-Golm, Germany; ^5^Max-Planck Partner Group at the Departamento de Biologia Vegetal, Universidade Federal de Viçosa, 36570-000 Viçosa, MG, Brasil; ^6^Departament de Biologia Vegetal, Universitat de Barcelona, Facultat de Biologia, 08028 Barcelona, Spain

**Keywords:** *Arabidopsis*, gene expression, metabolomics, nitrogen limitation, senescence, transcriptome.

## Abstract

Leaf senescence is a developmentally controlled process, which is additionally modulated by a number of adverse environmental conditions. Nitrogen shortage is a well-known trigger of precocious senescence in many plant species including crops, generally limiting biomass and seed yield. However, leaf senescence induced by nitrogen starvation may be reversed when nitrogen is resupplied at the onset of senescence. Here, the transcriptomic, hormonal, and global metabolic rearrangements occurring during nitrogen resupply-induced reversal of senescence in *Arabidopsis thaliana* were analysed. The changes induced by senescence were essentially in keeping with those previously described; however, these could, by and large, be reversed. The data thus indicate that plants undergoing senescence retain the capacity to sense and respond to the availability of nitrogen nutrition. The combined data are discussed in the context of the reversibility of the senescence programme and the evolutionary benefit afforded thereby. Future prospects for understanding and manipulating this process in both *Arabidopsis* and crop plants are postulated.

## Summary

Precocious senescence induced by nitrogen shortage can be reversed by N resupply. This study identifies the transcriptomic, metabolomic, and hormonal rearrangements underlying this process

## Introduction

Leaf senescence, the final stage of leaf development, is characterized by a decrease of photosynthetic activity, the degradation of proteins and other macromolecules, and the recycling of the liberated resources of the dying leaf for reuse in other growing parts of the plant, such as newly forming leaves, roots, developing seeds, or fruits (Gan and Amasino, 1997; Lim *et al.*, 2007; Gregersen *et al.*, 2013). Thus, although deteriorative by nature, leaf senescence is central for plant growth and reproductive success, important factors of plant fitness. In the course of leaf senescence, cells undergo dramatic changes in cellular metabolism, and leaves are converted from a nutrient sink into a nutrient source. This process is triggered by internal age-dependent factors, involves the action of phytohormones (Jibran *et al.*, 2013), and is profoundly modified by environmental parameters such as drought or nutrient deprivation (Guo and Gan, 2005; Wingler and Roitsch, 2008; Gregersen *et al.*, 2008; Guiboileau *et al.*, 2010).

The complex regulatory networks underlying the senescence programme are not yet fully understood, but recent analyses of global transcriptome changes imply massive reprogramming of gene expression during senescence (Buchanan-Wollaston *et al.*, 2005; van der Graaff *et al.*, 2006; Breeze *et al.*, 2011; Guo and Gan, 2012). While genes for photosynthetic proteins and chloroplast development are down-regulated during senescence, others, called senescence-associated genes (SAGs), are up-regulated. Among them are genes involved in the degradation and recycling of metabolic resources. In addition, many regulatory genes, including members of the WRKY and NAC transcription factor (TF) families and components of upstream signalling networks, are induced at the onset of senescence (Lim *et al.*, 2007; Balazadeh *et al.*, 2008; Breeze *et al.*, 2011). Furthermore, leaf senescence is controlled by epigenetic mechanisms (Ay *et al.*, 2009; Brusslan *et al.*, 2012; Humbeck, 2013). Recent analyses have shown that senescence can be induced by separate, but interacting regulatory pathways (Guo and Gan, 2012). Cross-talk between these pathways and other developmental and stress- and nutrient-sensing pathways in plants facilitates the optimization and fine-tuning of leaf senescence in response to changing environmental conditions.

The macronutrient nitrogen (N) is an abundant element in living organisms and a limiting factor for plant growth and development. During growth, roots take up inorganic N via nitrate, ammonium, or other forms, for example urea. In the plant cells, reduction of oxidized N leads to the formation of ammonium which to a large extent is assimilated into glutamine and glutamate; both amino acids, together with asparagine and aspartate, represent the main carrier forms of N transported via the phloem to sink tissues (Masclaux-Daubresse *et al.*, 2010). In photosynthetically active leaves the major part of the N (~80%) is bound in chloroplast proteins, with ribulose-1,5-bisphosphate carboxylase/oxygenase (Rubisco) representing the largest N storage (Kant *et al.*, 2011). N can be efficiently taken up from the soil, metabolized, and transported to developing leaves, enabling the development of the photosynthetic machinery and rapid tissue growth. However, when N supply is limited, plants have to economize on this valuable resource. One way to achieve this involves recycling N from older leaves during senescence for reuse in sink organs that require N for growth. Efficient N exploitation is most important when N supply and availability in the soil are low and therefore root N uptake is limited.

The inter-relationship between N availability and leaf senescence has long been known. Several reports demonstrate early leaf senescence under low-N conditions, and delayed leaf senescence when surplus N is supplied (Egli *et al.*, 1976; Schildhauer *et al.*, 2008; Gregersen *et al.*, 2013). Indeed, N availability is a major regulator of leaf senescence. Despite its importance for plant growth, not much is known about how the signal ‘N availability’ is implemented into the complex regulatory networks of leaf senescence.

In accordance with its central relevance for plant growth and development, recent transcriptome analyses revealed a large fraction of the *Arabidopsis thaliana* genome to be N responsive (Castaings *et al.*, 2011; Gutiérrez, 2012); however, our understanding of the molecular basis of N sensing and signalling underlying these responses and its connection to the senescence pathways is still fragmentary. Experimental evidence indicates that nitrate availability in roots can be sensed by the nitrate transporters NRT1.1 and NRT2.1 (Little *et al.*, 2005; Alvarez *et al.*, 2012), the MADS-box transcription factor ARABIDOPSIS NITRATE REGULATED1 (ANR1) (Zhang and Forde, 1998), the NIN-LIKE PROTEIN7 (NLP7) (Castaings *et al.*, 2009), and members of the LATERAL ORGAN BOUNDARY DOMAIN (LBD37/38/39) TFs which repress anthocyanin biosynthesis (Rubin *et al.*, 2009). Notably, N and carbon (C) metabolism are closely linked and the C/N ratio appears to function as a major signal affecting plant growth and development (Masclaux-Daubresse *et al.*, 2010; McAllister *et al.*, 2012). Glutamate, the product of the glutamine synthetase/glutamate synthase (GS/GOGAT) pathway and a major intercellular and inter-organ N carrier, also serves as a sensor of N status (Foyer *et al.*, 2003; Nunes-Nesi *et al.*, 2010). An increase of the C/N ratio, when high levels of sugars accumulate and only low levels of N are available, also induces leaf senescence (O’Hara *et al.*, 2013). In this case, trehalose 6-phosphate (T6P), the precursor of trehalose, serves as a signal for a high C level (Wingler *et al.*, 2012). T6P inhibits the SnRK1 protein kinase (Zhang *et al.*, 2009), a regulator of starvation stress responses in plants (Baena-González and Sheen, 2008). In addition, the levels of ceramides, lysolipids, and aromatic and branched chain amino acids, as well as stress-induced amino acids have been documented to accumulate and the nutrient ion content to become apparent in plants undergoing senescence (Watanabe *et al.*, 2013). Other known key players in the N-dependent regulation of plant development are phytohormones, which tune developmental processes at the whole-plant level and through interplay with N nutrition (Sakakibara *et al.*, 2006; Krouk *et al.*, 2011).

Several reports showed that N resupply can reverse the response to N starvation. For example, global expression analyses revealed that similar sets of genes are reacting to N starvation and N resupply in *Arabidopsis* seedlings, but often in opposite ways. The response to N resupply is fast, with changes in gene expression already within minutes (Scheible *et al.*, 2004). In addition, investigations of N transporters under N starvation and N resupply revealed distinct expression changes. For example, Gu *et al.* (2013) observed an up-regulation of the two *Zea mays* (corn) ammonium transporter genes *ZmAMT1;1a* and *ZmAMT1;3* when ammonium, but not nitrate, was resupplied after N starvation, indicating a substrate-specific regulation. Wang *et al.* (2012) analysed expression of the high-affinity urea transporter gene *OsDUR3* from rice (*Oryza sativa*) and found that it is generally up-regulated in roots during N depletion; in addition it can be specifically induced by the addition of urea, but not by ammonium, also indicating a substrate-specific regulation. In addition to effects on gene expression, Engelsberger and Schulze (2012) recently reported that N resupply leads to alterations in protein phosphorylation patterns within minutes after addition of N in the form of either nitrate or ammonium. These studies show that plants are able to respond specifically to both situations, namely N depletion and resupply of N.

An additional interesting observation is that in flag leaves of barley (*Hordeum vulgare*), senescence that is prematurely induced by low N can be stopped or even reversed when N is resupplied at the onset of senescence (Schildhauer *et al.*, 2008). At the molecular level, the resupply of nitrate had an effect on two marker genes of N metabolism, retarding the senescence-specific down-regulation of a plastidic glutamine synthetase (GS2) and the senescence-specific up-regulation of lysine-ketoglutarate reductase/saccharopine dehydrogenase (LKR/SDH). Resupply of nitrate or ammonium effectively reversed N starvation-induced leaf senescence, while the addition of urea did not. Similar to barley, precocious leaf senescence of N-limited *Arabidopsis* plants could be reversed by N resupply, but the wider molecular and metabolic rearrangements occurring during the reversal of senescence were not studied previously (Schildhauer *et al.*, 2008). The work presented here focuses on *Arabidopsis*. By using a combination of microarray-based transcriptome profiling, metabolite profiling, and hormone analyses, leaves which exhibit alterations in the senescence programme as a response to changes in N availability are compared, showing that both pathways are closely connected and the senescence programme is tuned by the N status. A set of key players of the N-dependent regulation of leaf senescence could be identified.

## Materials and methods

### Plant material

*Arabidopsis thaliana* (L.) Heynh. Col-0 plants were grown in hydroponic culture under a 16h/8h light (23 °C, 120 μE m^–2^ s^–1^)/dark (18 °C) regime. Seeds were first kept for 48h in the dark on moist filter paper at 6 °C and were then transferred to 0.2ml vials with a cut base filled with agarose in half-concentrated Hoagland medium (Tocquin *et al.*, 2003) [lower layer 0.5% agarose (w/v); upper layer 0.3% agarose (w/v)]. The vials were placed on top of the medium, enabling contact of the agarose with the medium [5mM KNO_3_, 1mM Ca(NO_3_)_2_, 0.5mM MgSO_4_, 0.15mM (NH_4_)_2_HPO_4_, 10 μM Fe-HBED, 9.6 μM H_3_BO_3_, 0.3 μM ZnSO_4_, 0.2 μM CuSO_4_, 0.14 μM Na_2_MoO_4_, 2 μM MnCl_2_, 0.1mM K_2_SiO_3_, 2mM MES; pH 5.7]. For N deprivation, all nitrate salts were replaced by chloride salts, and (NH_4_)_2_HPO_4_ was replaced by Na_2_HPO_4_. Leaves number 1 and 2 following the cotyledons were harvested at different time points after transfer of the seeds to the vials, immediately frozen in liquid N, and stored at –80 °C until use for RNA isolation and other analyses.

### Chlorophyll and PSII efficiency measurements

Chlorophyll was extracted with 80% (v/v) acetone, 19.5% (v/v) water, and 0.5% (v/v) concentrated NH_3_ solution (25% w/v) using an ultrasonic bath. After centrifugation, total chlorophyll content was analysed spectrophotometrically at 652nm and 750nm according to Goodwin (1976) [C_Chla+b_=27.8 (E_652_–E_750_) (mg l^–1^)].

Chlorophyll fluorescence measurements were carried out with an imaging PAM system (IMAGING-PAM Chlorophyll Fluorometer; Heinz Walz GmbH, http://www.walz.com) according to the method described by Baker (1991), using the saturation pulse method (Genty *et al.*, 1989). The leaves were kept for 30min in the dark before measuring the maximal photosystem II (PSII) quantum yield, *F*_v_/*F*_m_ (PSII efficiency).

### Transcriptome profiling

A 3 μg aliquot of quality-checked total RNA obtained from leaves (leaf nos 1 and 2) of plants grown in full N medium (19-, 23-, 23+3 h-, 26- and 29-day-old plants), N-free medium (23-, 23+3 h-, 26-, and 29-day-old plants), and plants subjected to N resupply for 3h [at 23 days after sowing (DAS); 23d3h sample] and 3 d (at 26 DAS; 26d3d sample) were processed for use in Affymetrix ATH1 microarray hybridizations. Three biological experiments were performed. Labelling, hybridization, and scanning were performed by ATLAS Biolabs (Berlin, Germany). Raw data (CEL files) obtained from RNA hybridization experiments were normalized with the affyPLM package from the Bioconductor software project (Gentleman *et al.*, 2004) using the GCRMA that uses the GC content of probes in normalization with RMA (robust multiple array average) and gives one value for each probe set instead of keeping probe level information (Wu *et al.*, 2004).

### Clustering of gene expression data

Cluster analysis and visualization of differentially expressed genes was achieved by the Short Time-series Expression Miner (STEM) software using default settings (Ernst and Bar-Joseph, 2006).

### GO enrichment analysis

To find overrepresented gene ontology (GO) terms in a set of genes, PLAZA 2.5 was used (van Bel *et al.*, 2012). After creating a personal account, a new experiment is set up for each profile and, after adding each profile’s genes, the GO enrichment can be directly calculated using the option ‘View the GO enrichment’. PLAZA 2.5 uses the GO terms included in the original annotation combined with a set of terms predicted through orthology projection; the enrichment is calculated using a hypergeometric distribution using all *Arabidopsis* genes as background; correction for multiple testing is automatically applied by multiplying *P*-values by the number of tests performed (Bonferroni correction).

### Motif enrichment analysis

The enrichment of known motifs was calculated using ATCOECIS (Vandepoele *et al.*, 2009); a list of genes present in each profile was entered online (section ‘Determine GO and motif enrichment using your genes as input’) to calculate the enrichment (using hypergeometric distribution with all *Arabidopsis* genes as background). Only motifs that occurred in at least 10% of the genes within a profile were considered to be relevant.

### *De novo* motif detection

Starting from a full set of 1kb *Arabidopsis* promoters (reverse complemented for genes on the negative strand), subsets were made for each profile. Using MEME (a component of MEMESuite), shared promoter motifs were detected in all subsets (dna, zoops, zero, or one motif per sequence), 10 motifs, 1 000 000 maximum input size while keeping motif length between 6bp and 12bp, using a background model based on all promoters). For each significant motif, the enrichment was calculated by dividing the frequency of the motif in the subset by the frequency of the motif in the complete set of promoters. The significance of the enrichment scores was calculated using the hypergeometric distribution, adjusting for multiple testing using Bonferroni correction (multiplying *P*-values by the number of tests).

Using the same workflow, MotifSuite was run; MotifSampler was used to detect the motifs (using a background model based on the full set of 1kb *Arabidopsis* promoters, 100 iterations were done, keeping two motifs in each run, running in single-strand mode). MotifRanking was used to filter the predictions, keeping only the top 10 motifs, which were mapped to the promoter subset and full set using MotifLocator with a significance threshold of 0.95 (other parameters for MotifSuite were left at their default values); the enrichment and significance were calculated as for MEMESuite

Using the annotation and genomic sequence, downloaded from PLAZA 2.5 (van Bel *et al.*, 2012), 1kb promoters were extracted for all dicot species (*Arabidopsis thaliana*, *Arabidopsis lyrata*, *Lotus japonicus*, *Medicago truncatula*, *Glycine max*, *Malus domestica*, *Fragaria vesca*, *Manihot esculenta*, *Ricinus communis*, *Populus trichocarpa*, *Carica papaya*, *Theobroma cacao*, and *Vitis vinifera*). Additionally, orthologous groups from PLAZA 2.5, generated by determining protein similarities using all-against-all BLAST (Altschul *et al.*, 1997) and clustering these similarities using OrthoMCL (Li *et al.*, 2003), were extracted. Orthologous promoter sequences for all genes containing a motif were extracted and the motif was mapped on these promoters using both MAST and MotifLocator. In the case where 30% or more of the orthologues contained the motif, according to one of the mapping tools, it is considered conserved. Note that motifs detected by MEME and MotifSampler/MotifRanking needed to be converted to formats compatible with MotifLocator and MAST, respectively; this was done using custom scripts. Motifs discovered in the different profiles were compared using TOMTOM (Gupta *et al.*, 2007), with a *q*-value threshold of 0.01.

### Hormone analyses

Phytohormones were measured by UPLC-MS/MS as described (Müller and Munné-Bosch, 2011).

### Metabolic profiling

Metabolite analysis was performed using ~75mg of fully expanded rosette leaves. The extraction, derivatization, standard addition, and sample injection were performed exactly as described previously (Lisec *et al.*, 2006). The gas chromatography–mass spectrometry (GC-MS) system was comprised of a CTC CombiPAL autosampler, an Agilent 6890N gas chromatograph, and a LECO Pegasus III TOF-MS running in EI+ mode. Metabolites were identified in comparison with database entries of authentic standards (Kopka *et al.*, 2005). Chromatograms and mass spectra were evaluated by using Chroma TOF 1.0 (Leco; http://www.leco.com/) and TagFinder 4.0 software (Luedemann *et al.*, 2008). Data presentation and experimental details follow recent recommendations (Fernie *et al.*, 2011).

## Results

### N deficiency-induced leaf senescence is reversed upon N addition in *Arabidopsis*

**Fig. 1.**
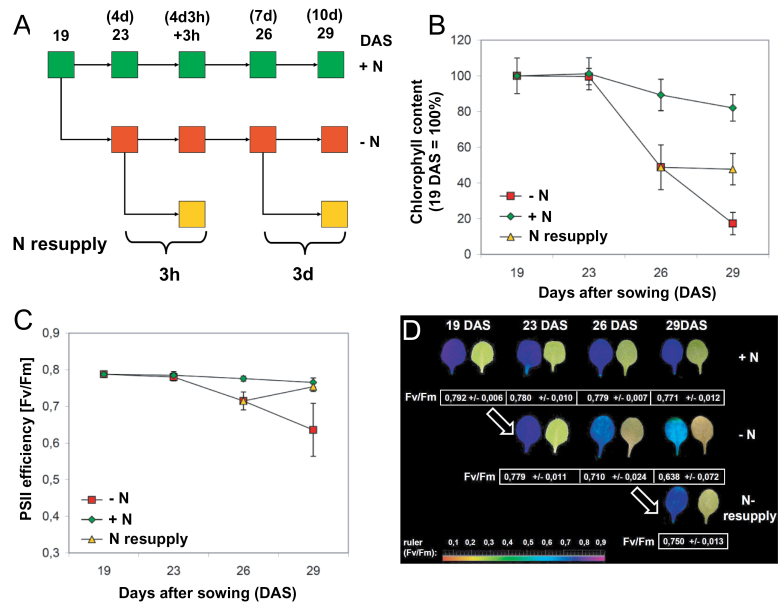
Experimental set-up. (A) Schematic representation of the experimental set-up: *Arabidopsis thaliana* plants were grown hydroponically in complete Hoagland liquid medium (+N medium). Plants were transferred to nitrogen-free medium (–N medium) 19 d after sowing (DAS). After 4 d and 7 d of growth on –N medium, subsets of plants were transferred back to complete medium (N resupply) for 3h or 3 d, respectively. (B) Chlorophyll content in leaves no. 1 and 2 of N-starved (–N), N-resupplied plants, and plants not starved for N (+N). Numbers on the *y*-axis indicate relative chlorophyll content. (C) Photosystem II (PSII) efficiency (*F*_v_/*F*_m_) in leaves no. 1 and 2 of N-starved (–N), N-resupplied plants, and plants not starved for N (+N). The chlorophyll fluorescence measurements were performed after 30min of dark adaptation. Measurements were performed on the total surface area of leaves. Data in (B) and (C) represent means ±SD (*n*=10). (D) Representative leaves and their PSII efficiency (*F*_v_ */F*_m_). For each day (DAS), pictures on the left represent the PSII efficiency in a chlorophyll fluorescence false-colour image, with a photograph of the same leaf on the right-hand side (see colour scale). Mean values of *F*_v_/*F*_m_ and standard deviations are displayed below each image (*n* ≥10 plants).

Reversal of leaf senescence in N-starved plants by N resupply has been reported for barley (Schildhauer *et al.*, 2008), but to the authors’ knowledge not in *A. thaliana*. Therefore, first an experimental condition that allowed reliable induction and then reversal of the progression of senescence was set up. *Arabidopsis* plants were grown hydroponically in complete Hoagland medium supplemented with N (7mM nitrate and 0.3mM ammonium; +N medium), with only roots submerged in medium. Nineteen DAS, plants were transferred to N-free medium (–N medium) and kept for another 10 d. Subsets of plants were fed again with N after 4 d of N limitation for 3h, or after 7 d of N limitation for 3 d. Control plants were cultured in +N medium throughout the entire growth period (i.e. 29 d). A schematic representation of the experimental set-up is shown in Fig. 1A. As a proxy for senescence progression, chlorophyll content and photosystem II (PSII) efficiency were determined in leaves number 1 and 2 (the first true leaves emerging after the cotyledons). Chlorophyll content and PSII efficiency did not change from days 19 to 23 (Fig. 1B, C), and remained unaffected in plants N starved for 4 d, indicating that dismantling of the photosynthetic apparatus did not start immediately after N depletion. As discussed below, metabolism already reacted to N depletion during the first 4 d of N depletion, with a clear decrease of most amino acids (see below) which is a proxy of N starvation (Foyer *et al.*, 2003; Nunes-Nesi *et al.*, 2010). At day 26, chlorophyll content started to decrease slightly in control plants kept in N-replete conditions for the entire growth period, potentially indicating an early phase of senescence; notably, however, PSII efficiency was not significantly affected in the control plants up to day 29. Upon extended growth in –N medium, the chlorophyll level declined strongly to ~50% of the original level at day 26, and to <20% at day 29 (Fig. 1B). Notably, when N-starved plants were fed again with N at day 26 (i.e. after 7 d of N limitation), no further loss of chlorophyll was observed, indicating that addition of N to senescing plants stopped the further progression of senescence. PSII efficiency largely mirrored the change of chlorophyll levels over the different treatments, albeit that overall changes were less prominent. Notably, PSII efficiency recovered to 100% in plants starved for 7 d when N was resupplied for 3 d (Fig. 1C), whilst it declined to ~80% of the original level upon extended N starvation (day 29). Figure 1D shows examples of leaves and false-colour images of PSII efficiency of control, N-depleted, and resupplied plants.

### Transcriptome analysis of N deficiency-induced leaf senescence and its reversal by N resupply

**Fig. 2.**
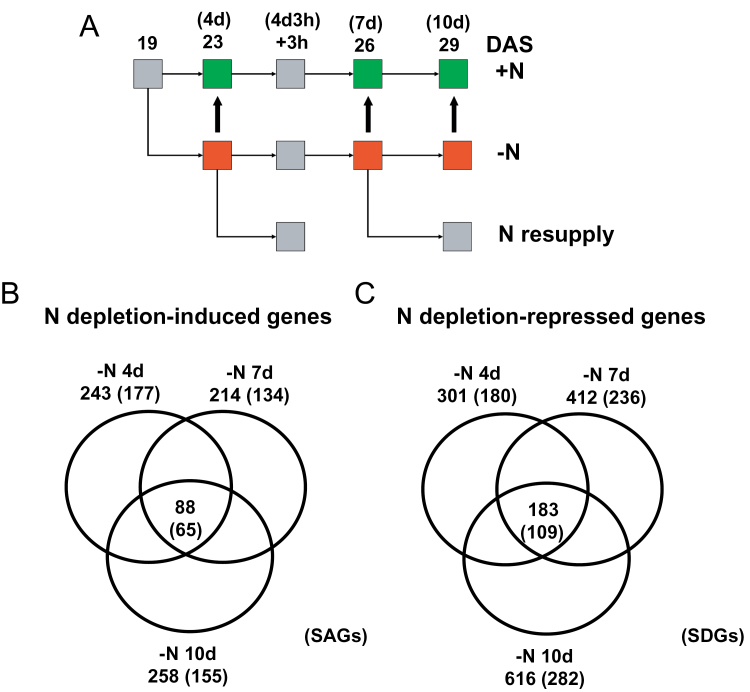
N depletion-induced and -repressed genes. (A) Schematic presentation of the sampling strategy. (B) and (C) Venn diagrams showing the numbers of significantly (B) up-regulated and (C) down-regulated genes that are uniquely or commonly regulated during nitrogen depletion. Numbers in parentheses represent numbers of SAGs (in B) and SDGs (in C) in each group. Thick arrows in (A) indicate samples that were compared for data shown in (B) and (C).

**Fig. 3.**
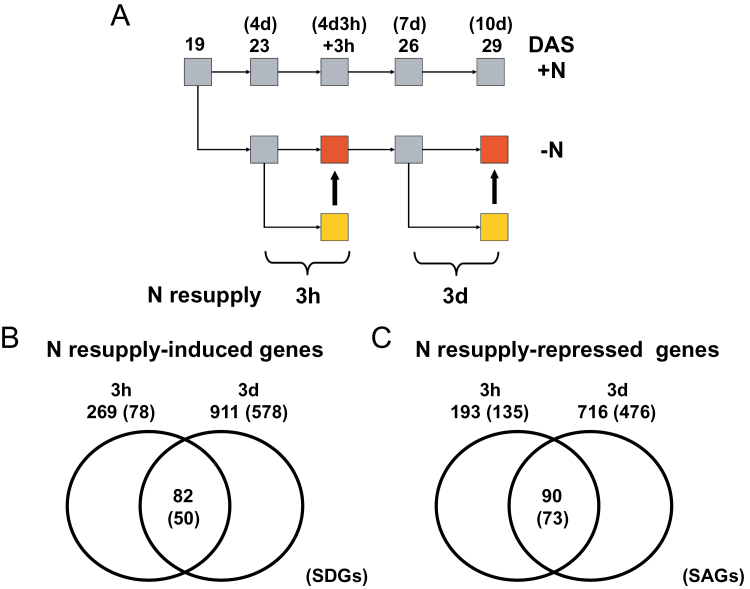
N resupply-induced and -repressed genes. (A) Schematic presentation of the sampling strategy. (B) and (C) Venn diagrams showing the numbers of significantly (B) up-regulated and (C) down-regulated genes that are uniquely or commonly regulated upon N resupply. Numbers in parentheses represent numbers of SDGs (in B) and SAGs (in C) in each group. Thick arrows in (A) indicate samples that were compared for data shown in (B) and (C).

To identify genes involved in N deficiency-induced leaf senescence and the reversal of the phenotype by readdition of N, transcriptome profiling was performed using Affymetrix ATH1 microarrays. RNA was isolated from leaves (nos 1 and 2) of plants grown in +N medium (time points: 19, 23, 26, and 29 DAS) or in –N medium (23, 26, and 29 DAS, corresponding to 4, 7, and 10 d, respectively, of N starvation). In addition, leaves were sampled from plants N starved for 4 d, but refertilized with nitrate for 3h (23 d+3h time point), and from plants N starved for 7 d, but refertilized with nitrate for 3 d (26 d+3 d time point); a schematic presentation of the sampling strategy is given in Figs 2A and 3A. Expression profiling was performed with RNA from three biological replicates for each time point (27 microarray hybridizations in total) and ratios of gene expression levels were calculated [log_2_ fold change (FC)].

First, it was of interest to identify genes affected by N limitation. To this end, the transcript profiles of plants deprived of N for 4, 7, and 10 d were compared and then compared with profiles from plants grown in +N medium. Considering a 3-fold expression change as the cut-off, 243, 214, and 258 genes were induced by N starvation after 4, 7, and 10 d, respectively; thus, in total, 447 genes responded positively to N starvation (Supplementary Table S1 available at *JXB* online). Eighty-eight genes were steadily induced from day 4 to day 10 after N removal. Notably, >60% of the N starvation-induced genes (at each examined time point) constitute known developmental senescence-up-regulated genes (SAGs; Buchanan-Wollaston *et al.*, 2005; van der Graaff *et al.*, 2006; Balazadeh *et al.*, 2008; Breeze *et al.*, 2011), indicating that N deficiency greatly affects expression programmes also acting during age-regulated leaf senescence (Fig. 2B). Expression of the established developmental senescence marker gene *SAG12* was highly (64-fold) induced 7 d after N limitation, further supporting the view that N limitation triggers a developmental senescence-related programme in plants at this stage (Supplementary Fig. S1 available at *JXB* online).

A total number of 301, 412, and 616 genes were repressed upon 4, 7, and 10 d of N removal, respectively (1184 genes in total; Supplementary Table S1 at *JXB* online). A total of 183 genes were steadily repressed from day 4 to day 10 of N starvation; 180, 236, and 282 genes of the genes repressed at 4, 7, and 10 d, respectively, are known senescence-down-regulated genes (SDGs; Buchanan-Wollaston *et al.*, 2005; Breeze *et al.*, 2011) (Fig. 2C).

Next, in order to identify genes undergoing expression changes upon readdition of N, the transcript profiles of 4 d and 7 d nitrate-starved plants were compared with those of plants resupplied with N for 3h (after 4 d of N deficiency) and 3 d (after 7 d of N deficiency). The data revealed that expression of 269 genes (including 78 SDGs) and 911 genes (including 578 SDGs) was induced, while expression of 193 genes (including 135 SAGs) and 716 genes (including 476 SAGs) was repressed upon resupply of N for 3h and 3 d, respectively (Fig. 3). As shown in Supplementary Fig. S1 at *JXB* online, expression of *SAG12* was highly reduced (~500-fold), while expression of *SEN1*, an N depletion-repressed gene (see cluster analysis below), was highly induced after 3 d of N resupply. Taken together, the gene expression data indicate extensive reversal of the molecular senescence phenotype 3 d after readdition of N, which is in accordance with the reversal of photosynthetic activity quantified by measuring PSII efficiency (see above). Interestingly, already 3h after resupply of N, plants showed a clear response at the transcriptome level, including down-regulation of SAGs and up-regulation of SDGs.

### Comparison of gene expression patterns of N-regulated genes and genes affected during *Botrytis cinerea*- and dark-induced senescence

**Fig. 4.**
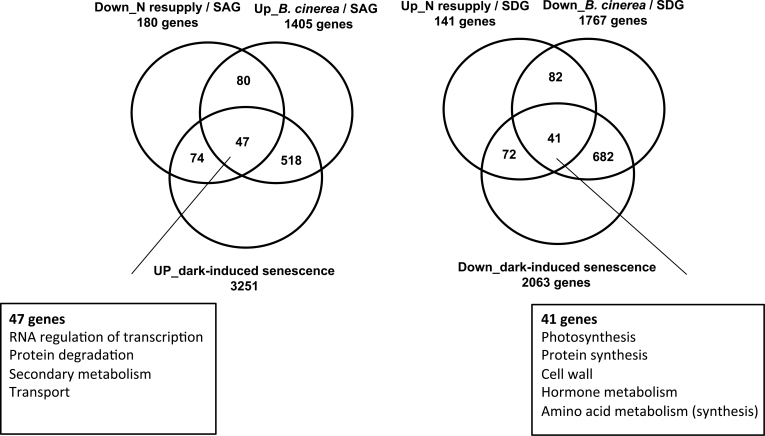
Number and function of genes commonly and differentially expressed upon N resupply, *Botrytis cinerea* infection, and dark-induced senescence. The numbers of genes are shown in the Venn diagrams.

Infection with the necrotrophic fungus *Botrytis cinerea* and dark treatment have been shown to induce leaf senescence (Weaver and Amasino, 2001; Lin and Wu, 2004; Swartzberg *et al.*, 2008; Windram *et al.*, 2012). Comparison of gene expression patterns induced by *B. cinerea* infection or dark treatment with developmental leaf senescence revealed considerable overlap of genes induced or repressed, respectively, by the different treatments (Lin and Wu, 2004; Buchanan-Wollaston *et al.*, 2005; Parlitz *et al.*, 2011; Windram *et al.*, 2012). To identify genes with common and distinct expression patterns in response to N limitation or N resupply and other stresses that affect senescence, genes differentially expressed upon changed N resupply (this report) were compared with genes affected during *B. cinerea*- and dark-induced senescence. For N supply-affected senescence, SAGs and SDGs showing a change in expression after 3h (by at least 2-fold) and 3 d (by at least 3-fold) of N resupply were considered; these were 180 SAGs down-regulated by N resupply and 141 SDGs up-regulated by N resupply. For *B. cinerea*-induced senescence, the data reported by Windram *et al.* (2012) were used and genes differentially expressed during both age-dependent senescence and *B. cinerea* infection were selected. For dark-induced senescence, data reported by Lin and Wu (2004) were chosen, focusing on genes differentially expressed after 5 d of dark treatment. A total of 47 and 41 genes, respectively, were commonly induced and repressed in all three senescence data sets (Fig. 4). Genes up-regulated in all three senescence data sets are mainly involved in RNA regulation of transcription (including the NAC TFs *ANAC019*, *ANAC032*, *ANAC047*, *ANAC055*, and *ANAC092*/*ORE1*), protein degradation, secondary metabolism, and transport activity (Fig. 4; Supplementary Table S2 at *JXB* online). The fact that *ANAC092*/*ORE1* is among the commonly up-regulated genes is in accordance with its proven central role as a positive regulator of senescence (Kim *et al.*, 2009; Balazadeh *et al.*, 2010; Trivellini *et al.*, 2012; Matallana-Ramirez *et al.*, 2013; Rauf *et al.*, 2013). Genes down-regulated in all three senescence data sets are mainly involved in photosynthesis, protein synthesis, cell wall modification, and hormone and amino acid metabolism (Fig. 4; Supplementary Table S3 available at *JXB* online), but also include the transcription regulators *TCP21* (At5g08330; also called *CHE* for *CCA1 Hiking Expedition*; Pruneda-Paz *et al.*, 2009) and *B-BOX DOMAIN PROTEIN14* (*BBX14*; *At1g68520*). TCP21 has recently been shown to interact with the transcriptional regulator TIMING OF CAB EXPRESSION1 (TOC1) to regulate the expression of the core-clock regulator *CIRCADIAN CLOCK ASSOCIATED1* (*CCA1*) (Pruneda-Paz *et al.*, 2009); its down-regulation in all analysed senescence data sets may indicate that control over the core circadian clock is lost during senescence. The biological role of *BBX14* has not been reported yet; it is of note, however, that *BBX14* expression is up-regulated by cytokinin treatment (Arabidopsis Hormone Database; http://ahd.cbi.pku.edu.cn), consistent with the well-known ability of this phytohormone to inhibit the progression of senescence (Gan and Amasino, 1996; Zwack and Rashotte, 2013).

A total of 101 genes were specifically differentially expressed during N limitation-induced senescence (with 73 genes down-regulated and 28 up-regulated, respectively, upon N resupply) (Supplementary Table S4 at *JXB* online). Of the genes specifically down-regulated during N resupply, several encode transcription regulators including the basic helix–loop–helix TFs *bHLH42* and *BIGPETALp* (*BPEp*, known to limit *Arabidopsis* petal growth by influencing cell expansion), a RWP-RK family protein (*At2g43500*), the bZIP TF *TGA7* previously shown to be induced by N limitation, and the two MYB transcription factors *PAP1* (*MYB75*) and *PAP2* (*MYB90*) involved in the regulation of anthocyanin biosynthesis. Additionally, genes involved in flavonoid biosynthesis including *F3’H* (*At3g51240*), *DFR* (*At5g42800*), *CHS* (*At5g13930*), and *UF3GT* (*At5g54060*), and phenylpropanoid biosynthesis (*At4g30470*, *At5g67160*), as well as genes encoding enzymes involved in N metabolism, including glutamine synthases GLN1;4 (*At5g67160*) and GLN1;1 (*At5g37600*), and genes encoding nutrient transporters such as *At1g12940* (NTR2.5 nitrate transporter), *At5g63850* (AAP4, an amino acid transporter), *At1g69480* (EXS, a phosphate transporter), and *At5g50800* (SWEET13, a sugar transporter), were repressed upon N resupply. The N resupply-specific reduction in the expression of genes involved in secondary metabolism (flavonoids and phenylpropanoids) is in accordance with previous findings which indicated a role for N in flavonoid and anthocyanin biosynthesis (Bongue-Bartelsman and Phillips, 1995; Stewart *et al.*, 2001; Strissel *et al.*, 2005; Peng *et al.*, 2007). Among genes specifically up-regulated during N resupply are genes involved in ‘RNA regulation of transcription’, ‘transport activity’, and ‘cellular signalling’.

### Cluster analysis of differentially expressed genes

**Fig. 5.**
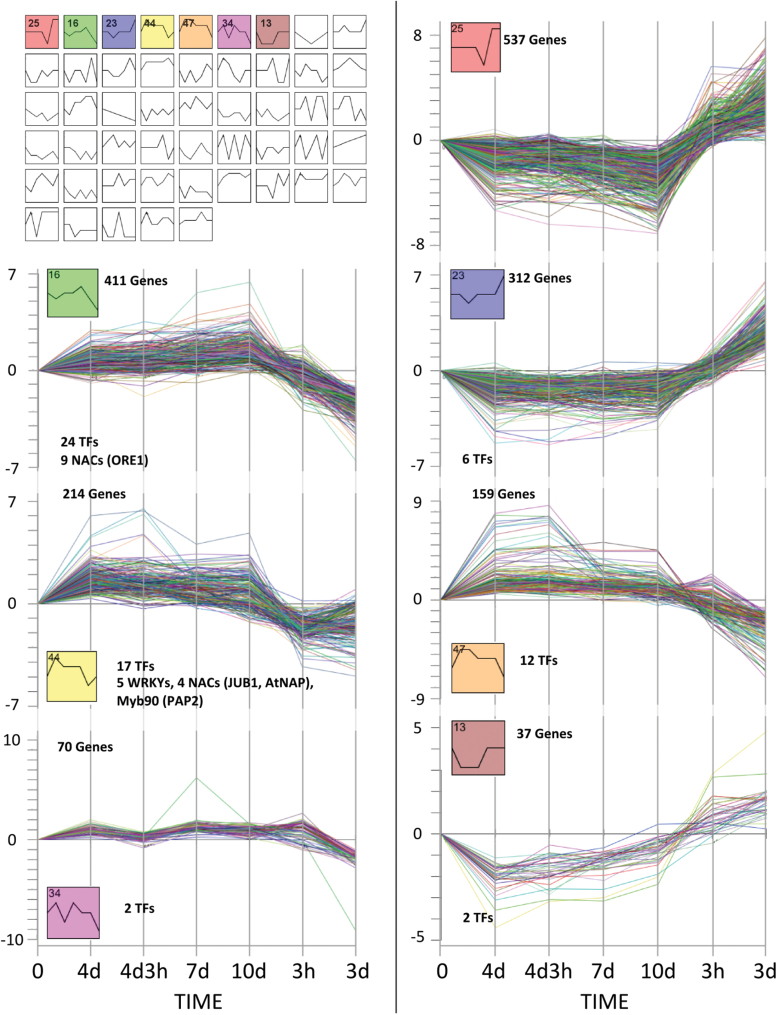
N depletion- and N resupply-responsive transcripts grouped according to temporal expression profiles. Differentially expressed genes obtained from Affymetrix ATH1 microarray-based transcriptome studies were divided into seven distinct significant temporal profiles, using STEM software (Ernst and Bar-Joseph, 2006). Each of the profiles is represented as a different plot, with mean expression ratios (log_2_) for each of the assigned transcripts at each time point. The presence of TFs in each profile is indicated.

Next, STEM (Short Time-series Expression Miner) was used to cluster and visualize microarray expression profiles of all genes differentially expressed upon N depletion and N resupply at any given time point of the study, comprising 1742 genes in total. STEM generates a series of potential profiles with respect to the direction and magnitude of expression, after which enrichment of clusters is determined by comparing the distribution of observed groups with those expected in a random permutation (Ernst *et al.*, 2005; Ernst and Bar-Joseph, 2006). Based on the expression patterns, profiles were classified into 50 categories. Figure 5 shows gene expression profiles for the seven clusters (clusters 13, 16, 23, 25, 34, 44, and 47) found to be significant (*P*-value ≤0.05) out of 50 possible clusters. Thirty-one percent of the total number of genes clustered belong to cluster 25, ~24% to cluster 16, ~18% to cluster 23, ~12% to cluster 44, ~9% to cluster 47, ~4% to cluster 34, and ~2% to cluster 13 (Fig. 4; Supplementary Table S5 at *JXB* online).

Visual inspection of the clusters revealed a clear response in expression for the majority of the genes after N resupply. Clusters 16, 44, 34, and 47 contain 411, 214, 70, and 159 genes, respectively. Overall, expression of genes in these clusters increased with N limitation and returned to the initial level or declined further when N was resupplied. Several genes involved in the regulation of transcription (TFs) were present in these groups. Cluster 16 includes 24 TF-encoding genes; of those, 19 are known SAGs. Among the senescence-associated TFs, members of the NAC family were over-represented in this group (9 out of 19), and the key positive senescence regulator *ORE1* (*ANAC092*) was among them. Cluster 44 includes 17 TFs, 16 of which are known SAGs. Examples include *PAP2* (MYB90), several members of the WRKY family (such as *WRKY33*, *44*, *18*, *45*, and *46*), and *AtNAP* (*ANAC029*) and *JUB1* (*ANAC042*), members of the NAC family playing key roles in the regulation of leaf senescence (Guo and Gan, 2006; Wu *et al.*, 2012). AtNAP has recently been reported to play a role in leaf senescence partly via control of the expression of *SAG113*, its direct target gene. *SAG113* is an early senescence-induced gene encoding a protein phosphatase (PP) 2C localized in the *cis*-Golgi apparatus. Its expression is induced by abscisic acid (ABA) and it functions as a negative regulator of ABA signalling during leaf senescence (Zhang and Gan, 2012; Zhang *et al.*, 2012). JUB1 negatively regulates senescence by dampening the cellular H_2_O_2_ level (Wu *et al.*, 2012).

Of the TFs included in clusters 47 and 34, nine (out of 12) and two (out of two), respectively, were SAGs. Clusters 25 (537 genes), 23 (312 genes), and 13 (37 genes) include genes whose expression decreased upon N limitation and returned to the initial level or increased further upon readdition of nitrate. Clusters 25 and 23 contained many genes involved in photosynthesis; *SEN1* (Supplementary Fig. S1 at *JXB* online) is included in cluster 25 (Supplementary Table S5 available at *JXB* online). Cluster 47 included genes up-regulated during N depletion and down-regulated by N resupply. Next, the most significant co-expression clusters were selected for subsequent functional group and motif analysis.

### GO enrichment analysis

Genes included in the expression profiles presented in Fig. 5 were imported to different workbench experiments in PLAZA 2.5 (van Bel *et al.*, 2012). As such, GO terms that are significantly over-represented in the different profiles could be detected. By comparing over-represented terms describing the cellular component, it can be observed that the different expression profiles appear to be associated with different compartments of the cell. Profile 13 seems to be correlated with genes encoding proteins that are targeted to the cell wall, while profiles 23 and 25 are strongly enriched with chloroplast genes. Profile 34 is enriched for genes encoding nuclear and mitochondrial proteins, as is profile 16, albeit to a smaller extent. This result indicates that different components of the cell have considerably different responses as N starvation continues.

Besides the most prevalent cellular localizations of the proteins encoded by the genes in these expression profiles, the enrichment study also provides an insight into the biological processes being regulated. Only profile 16 is enriched for genes previously associated with leaf senescence. Though the N deficiency seems to trigger various stress or stimulus responses, profiles 13, 25, 44, and 47 all show enrichment for terms describing a ‘response to … stress’ or ‘response to … stimulus’. The remaining profiles 23 and 34 were enriched for GO terms related to photosynthesis and mitochondrial biological processes, respectively (a full overview of all enriched terms is given in Supplementary Table S6 at *JXB* online).

### Motif enrichment analysis and *de novo* motif detection

As for the GO enrichment analysis, the genes included in the different profiles were analysed using ATCOECIS (Vandepoele *et al.*, 2009). This tool identifies an over-representation of known motifs derived from AGRIS (Palaniswamy *et al.*, 2006) and PLACE (Higo *et al.*, 1999), or conservation with the poplar genome in the 1kb promoter region of the analysed genes. Here, profile 13 was found to contain several motifs, though only one was classified (a glutenin box). Profile 34 was found to show over-representation of various motifs, most notably AAACCCTAA, a telo-box motif known to be conserved in known plant eEF1A (elongation factor) genes. The well-known ABRE motif (abscisic acid responsive element), which is hallmarked by a ACGT core sequence and known to be present in promoters of genes responsive to reactive oxygen species (Petrov *et al.*, 2012), was observed in several over-represented motifs in profiles 25 and 44. More specifically, profile 44 contains motifs with the G-box (CACGTG), a light-responsive *cis*-element (Menkens *et al.*, 1995; Terzaghi and Cashmore, 1995). Finally, profile 47 contains several motifs, which are, however, currently unknown. It is also quite common for ABRE, DRE, CArG, and G-box motifs to occur in other profiles, though at a frequency below the set threshold; for example, profile 16 contains various types of such motifs but not above the set requirement that they need to occur in 10% of the genes in the set; all enriched motifs found using ATCOECIS are available in Supplementary Table S6 at *JXB* online.

Motifs present in the JASPAR database (Bryne *et al.*, 2008) were also mapped to all *A. thaliana* 1kb promoter regions and the enrichment calculated for the different STEM profiles. This revealed a significant Dof2 binding site in profile 16, which in previous studies in maize (*Z. mays*) has been linked to carbon metabolism (Yanagisawa, 2000). By mapping the JASPAR motifs, an additional EmBP-1 binding site (a G-box-containing motif) was found in profile 23 and the presence of this motif in profiles 25 and 44 was confirmed. In profile 44, two additional binding sites were found for bZIP910 and TGA1A, which bind ATGACGT and TACGTCA consensus sequences, respectively. Both leucine zipper types of TFs are known to bind G-box-like sequences (Schindler *et al.*, 1992).

**Fig. 6.**
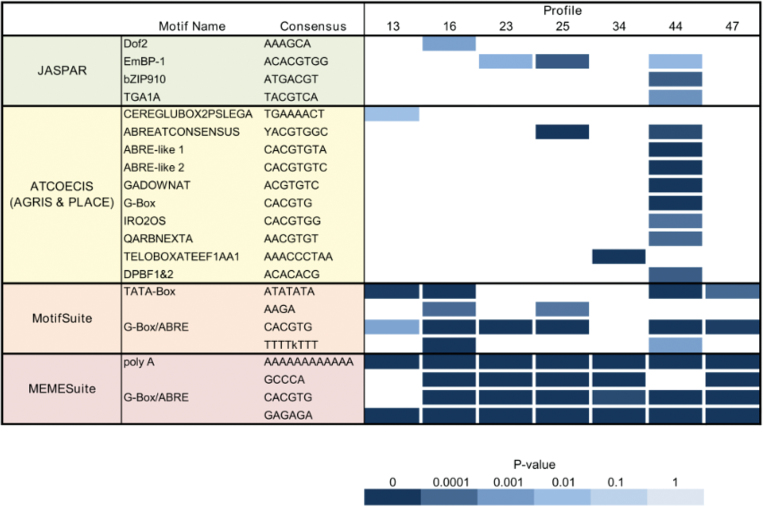
Regulatory sequences associated with groups of co-expressed genes. Significantly over-represented (*P*<0.01) motifs from JASPAR and ATCOECIS as well as motifs discovered *de novo* using MotifSuite and MEMESuite are shown for the seven STEM clusters. Note that the *de novo* detected motifs were also subjected to an enrichment test and only motifs present in multiple clusters are included in this image (a full overview is given in Supplementary Table S7 available at *JXB* online). In particular, motifs that contain the light-responsive G-box and ABRE sequences occur frequently.

Additionally, *de novo* motif prediction was performed on the 1kb promoters of the genes in each profile. This was done using both MEMESuite (Bailey *et al.*, 2009) and MotifSuite (Claeys *et al.*, 2012); in both cases, a background model was generated using all 1kb promoters of the *A. thaliana* genome. For MEMESuite, after the initial detection of the motifs using MEME, MAST was used to map these motifs back to the promoters of the profile and the entire set of 1kb promoters. The numbers of hits in both the profile and the *Arabidopsis* genome were used to calculate the significance using a hypergeometric distribution corrected for multiple testing. The same workflow was followed for MotifSuite, where the detection was done using MotifSampler and MotifRanking, while the mapping was done using MotifLocator. To see whether the motifs found are conserved, they were mapped on promoters of orthologous genes using both MAST and MotifLocator; motifs that reoccurred in at least 30% of the orthologous genes were considered to be evolutionarily conserved (a detailed output of all *de novo* detected motifs is given in Supplementary Table S7 at *JXB* online). This confirmed the G-box motif in profiles 23, 25, and 44, while revealing similar motifs in all other profiles except 13. As the plant hormone ABA plays an important role in plant senescence (e.g. Yang *et al.*, 2011; Zhang and Gan, 2012), the frequent appearance of the ABRE motif (or more specific longer variants) is not surprising. MotifSuite revealed a TATA-box-containing motif in profiles 13, 16, 44, and 47, which is reported to be, like the G-box, involved in light-dependent gene expression in plants (Kiran *et al.*, 2006). Additionally, *de novo* motifs that occurred in more than one profile along with the results from ATCOESIS and JASPAR are shown in Fig. 6.

### Hormonal profile of N deficiency-induced leaf senescence and its reversal by N resupply

It was of interest to know the effect of N deficiency on phytohormone levels. Thus, the concentrations of various hormones including ABA, jasmonic acid (JA), gibberellin 4 (GA_4_), salicylic acid (SA), and zeatin riboside (ZR) were determined in N-deficient plants and were compared with plants grown in full N medium (–N compared with +N) or in plants subjected to readdition of N (N resupply compared with –N). As shown in Supplementary Fig. S2 at *JXB* online (and Supplementary Table S8, available at *JXB* online), the level of ZR, one of the most active and ubiquitous forms of the naturally occurring cytokinins, declined with age in plants grown in full N medium. Its level also had a tendency to decrease after 4 d and 7 d of N removal, although this decrease was not significant. The level of ZR slightly increased within 3h after N resupply and fully recovered after 3 d. Cytokinin has been widely discussed as a hormone that has a regulatory function in senescence. Cytokinin levels are reduced in senescing leaves, whereas the exogenous application of cytokinins or elevation of cytokinin biosynthesis in transgenic plants delays senescence (Smart *et al.*, 1991; Gan and Amasino, 1995). An interaction between cytokinin and N signalling has also been widely discussed; a link between cytokinin content and N supply has, for example, been reported in barley (Samuelson and Larsson, 1993), tobacco (Singh *et al.*, 1992), *Urtica dioica* (Wagner and Beck, 1993), sunflower (Salama and Wareing, 1979), and maize (Takei *et al.*, 2001). In *Arabidopsis*, the levels of both isopentenyl- and zeatin-type cytokinins were higher in roots and shoots of seedlings grown at high concentrations of nitrate (10mM), indicating that cytokinins act as an N status signal (Kiba *et al.*, 2011). Moreover, accumulation of cytokinins was suppressed in *Arabidopsis* mutants lacking a functional *IPT3* gene (a nitrate-inducible gene encoding an enzyme that catalyses the initial step of cytokinin biosynthesis). In contrast to cytokinins, ABA levels significantly increased after 4 d and 7 d of N deficiency, which is in agreement with previous studies (Chapin, 1990; Peuke *et al.*, 1994), but ABA levels did not recover after N resupply. This indicates that senescence reversion by N resupply is mediated by cytokinins, but not by ABA.

JA content decreased during senescence and during N deprivation, and increased after N resupply. A significant increase after N resupply was also observed for the SA level. Both phytohormones, JA and SA, play key roles as mediators of plant stress responses, and the JA and SA signalling pathways are also active in the control of gene expression during developmental senescence (Morris *et al.*, 2000; He *et al.*, 2002). Thus, increases in JA and SA after N resupply may be indicative of improved defence. No significant change upon N deficiency or N resupply was observed for the level of GA_4_ at any given time point.

Ethylene (ET) promotes senescence in an age-dependent manner (Grbic and Bleecker, 1995). Two key enzymes are involved in ET biosynthesis, namely 1-aminocyclopropane-1-carboxylic acid (ACC) synthase (ACS), which converts *S*-adensoylmethionine to ACC, and ACC oxidase (ACO) which then metabolizes ACC to ET (Yang and Hoffmann, 1984). Expression of *ACS2*, *6*, *7* and *ACO2*, *3*, *4* is enhanced during senescence. Here it was observed that expression of *ACS6* was enhanced after 4 d (and 4 d+3h) of N deficiency and reduced at 3h after N resupply. Expression of *ACO2* and *ACO4* increased upon N deprivation and was significantly down-regulated after 3 d of N resupply. The observed changes in gene expression further confirmed that readdition of N reversed the senescence phenotype induced by N deficiency.

### Metabolome analysis of N deficiency-induced leaf senescence and its reversal by N resupply

**Fig. 7.**
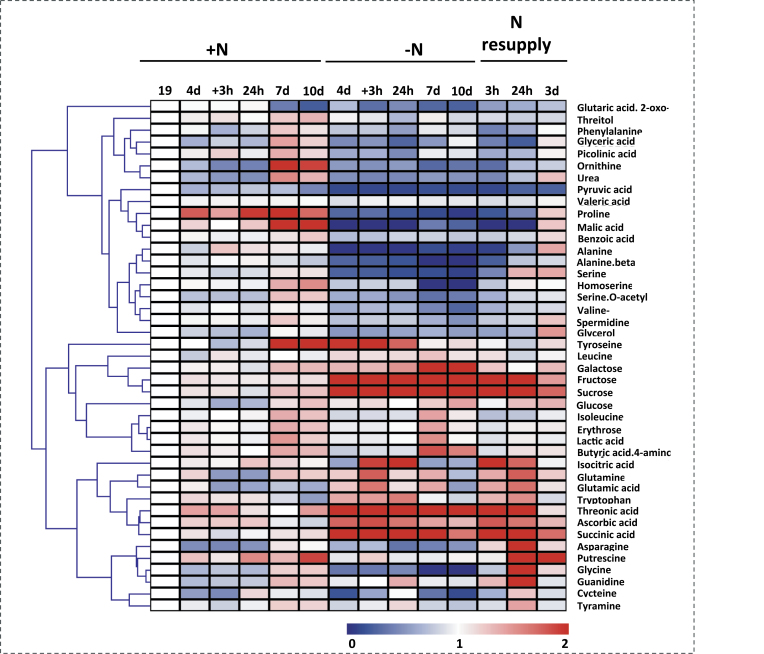
Primary metabolite profiling of N deficiency-induced leaf senescence and its reversal by N resupply. Hierarchical average linkage clustering of all detected primary metabolites. For every metabolite, the metabolic content of the control sample harvested at 19 DAS was considered as 1 and the metabolic content of all other samples at any given time point (+N, –N, and N resupply) normalized to that. Metabolic ratios: blue, minimum (between 0 and 1); red, maximum (between 1 and 2); see also Supplementary Table S9 available at *JXB* online.

To follow the repertoire of metabolic changes that occur in leaves in response to N deprivation, extensive metabolic profiling was carried out using an established GC-MS metabolic profiling protocol (Lisec *et al.*, 2006). These studies revealed considerable changes in the levels of a wide range of organic acids, amino acids, and sugars (Fig. 7; Supplementary Table S9 at *JXB* online; the full data sets from these metabolic profiling studies are additionally available as Supplementary Table S10 at *JXB* online). Thus, relatively minor changes, including reductions of glutamate and tryptophan as well as 2-oxoglutarate and pyruvate levels, were observed in plants grown in full N medium (Supplementary Table S9 available at *JXB* online). At the same time, significant but relatively small increments in the amino acids glycine, homoserine, proline, isoleucine, tyrosine, and ornithine, as well as in the sugars galactose, glucose, erythrose, and sucrose were observed in leaves between 19 and 29 DAS (10 d sample). Increases in the organic acids malate, γ-aminobutyric acid (GABA), threonate, ascorbate, and benzoate were also observed (Supplementary Table S9 at *JXB* online). These combined changes are suggestive of a respiratory switch from predominantly sugar- to predominantly protein-based respiration, as has previously been documented (Watanabe *et al.*, 2013).

The total amino acid content typically decreases when N is limiting (for a review, see Foyer *et al.*, 2003; Nunes-Nesi *et al.*, 2010, and references therein). Here, the situation is similar and the N removal treatment led to a rapid decline in the levels of most amino acids, exceptions being the levels of glutamate and glutamine as well as tryptophan and tyrosine which transiently increased rapidly after N removal but after 10 d without N were strongly reduced (Supplementary Table S9 at *JXB* online). This would imply that the reduced growth normally observed under N limitation (e.g. Tschoep *et al.*, 2009) is not triggered by decreased levels of all amino acids, but rather by changes in the regulation of central metabolism, as observed by the increased levels of glutamate and glutamine. Surprisingly, in contrast to the situation normally observed in N-limited plants (Foyer *et al.*, 2003; Lemaitre *et al.*, 2008), the glutamine/glutamate ratio was not altered in the present study. Similar to the situation described above for the amino acids, the levels of the majority of organic acids were reduced during N starvation (Supplementary Table S9 at *JXB* online). The strong decrease in pyruvate levels under N limitation could contribute to the large decrease in alanine during the light period. In contrast, the sugars galactose, fructose, and sucrose were significantly increased. In a similar vein, the levels of GABA, threonate, ascorbate, and succinate were also increased during N starvation (Supplementary Table S9 at *JXB* online).

To understand how metabolism responds to N resupply along with the reversal of senescence, the metabolite profiles were analysed following 3h, 24h, and 3 d of N resupply. Although it was observed that 3h of N resupply did not fully reverse the majority of the changes observed during N depletion, significant increases in the levels of the amino acids β-alanine, glycine, serine, homoserine, cysteine, valine, and acetylserine, as well as of the organic acid glycerate were observed. Moreover, after only 3h, the levels of the amino acids asparagine, glutamine, glutamate, and tryptophan and the organic acid GABA, as well as the sugar glucose returned to values similar to those observed before the N removal (Supplementary Table S9 at *JXB* online). It should be mentioned that 24h after N resupply the levels of homoserine, cysteine, tyrosine, ornithine, galactose, and benzoate had returned to the initial values observed in N-rich plants. Notably, the levels of almost all metabolites determined here returned to values observed before the N limitation after 3 d of N resupply. From a metabolic perspective, it indicates that this time was enough for plants to recover fully.

## Discussion

Leaf senescence is a developmentally controlled process that is typically activated only after the leaf has gone through a series of processes—including, for example, cell proliferation, chloroplast formation, and cell expansion—before it finally reaches a stage where further growth stops and senescence is initiated. However, although leaf formation and senescence follow a distinct developmental programme, the environment can have a profound effect on both the initiation and progression of senescence, and N limitation is one such a trigger (Diaz *et al.*, 2006; Vanacker *et al.*, 2006; Masclaux-Daubresse *et al.*, 2010; Kant *et al.*, 2011). Although N starvation induces senescence in plants, it has also been reported that senescence is reversible in some cases after resupply of N (Girardin *et al.*, 1985; Schildhauer *et al.*, 2008). However, the underlying molecular and metabolic mechanisms have not been studied in any detail in the past. Here, *A. thaliana* was used to establish an experimental set-up that allowed first senescence to be induced by N removal from well-fertilized plants, and then senescence to be reversed by N resupply. N starvation triggered a decline in leaf chlorophyll content after ~5 d, and this level was reduced to about half within 7 d of starvation (Fig. 1B). Notably, however, when N was resupplied on day 7 of starvation, the chlorophyll content remained stable until the last day of the experiment, while the chlorophyll content declined further upon continued starvation (Fig. 1C). Similarly, PSII efficiency decreased during N starvation, but even recovered to a normal level upon N resupply at day 29. In accordance with the model that senescence is induced after 7 d of N starvation (at day 26), expression of the senescence marker gene *SAG12* was strongly enhanced compared with plants kept under N-replete condition. Notably, *SAG12* expression strongly declined again when N was resupplied (Supplementary Fig. S1A at *JXB* online). Finally, the level of ZR, one of the most active naturally occurring cytokinins, had the tendency to decline in N-starved plants, concomitant with the development of senescence, but regained normal levels after 3 d of N resupply. Typically, cytokinins retard senescence (Lim *et al.*, 2007; Kiba *et al.*, 2011), in accordance with the rise of ZR levels seen here after N resupply. Thus, the data clearly demonstrate that N limitation-induced senescence in *Arabidopsis* can be stopped and even reversed to a large extent by N resupply, indicating that *Arabidopsis* retains its capacity to signal N status well into the senescence phase. The fact that an extensive but not full physiological recovery was achieved upon N resupply may be explained by the progressing age of the plants during the time course of the experiment and the accumulation of age-related factors, as suggested by Jibran *et al.* (2013). Although the cellular or molecular nature of such age-related factors remains elusive as present, N starvation may perhaps speed up their accumulation.

The experimental set-up was designed to study the reversal of N deficiency-induced senescence processes and thus differs from previous experiments, which often used seedlings not undergoing senescence processes, or used set-ups where the reversal was not specifically addressed. Scheible *et al.* (2004) identified rapid (within 3h) transcriptomic responses of 7-day-old seedlings grown in N-replete liquid medium to low-N treatment. Their experimental set-up (young seedlings, short treatment times) did not allow a look at senescence-related effects. Similarly, Wang *et al*. (2000, 2003) analysed seedlings grown in liquid medium to identify nitrate-responsive genes within a time span of up to 2h after the change of nitrate concentration. The physiological constraint in these experiments is that whole plantlets including leaves directly experience the change in N supply, which normally does not happen in soil-grown plants where changes in N nutrition first occur in the roots and then in the leaves. More recent reports therefore used hydroponic culture systems with only roots submerged in growth medium to test the effect of altered N nutrition. Using Affymetrix ATH1 microarrays, Patterson *et al.* (2010) analysed rapid transcriptional responses (up to 8h) in roots in nitrate- or ammonium-supplied *Arabidopsis* plants. Vidal *et al.* (2013) employed Illumina high-throughput sequencing (RNA-seq) to characterize poly(A)^+^ and small RNA fractions in response to short-term (2h) nitrate treatment in *Arabidopsis* roots. As leaves were not analysed and treatment times were short, no conclusions with respect to leaf senescence can be drawn from these studies.

Another study analysed the effect of chronic N stress by growing plants for up to 3 weeks at 0.3mM (severe N limitation) or 1mM (mild N limitation), while control plants were grown at 3mM (N sufficient) nitrate concentration (Bi *et al.*, 2007). Global expression profiling revealed only 52 genes to be differentially expressed in shoots of N-replete-grown plants compared with plants mildly N stressed, while the comparison of plants grown under optimal nitrate concentration (3mM) with those stressed chronically at 0.3mM nitrate identified 461 differentially expressed genes. Severe N limitation caused the down-regulation of many primary metabolism and N assimilation genes, as well as genes involved in photosynthesis. Genes encoding enzymes involved in protein degradation were induced, including the senescence marker gene *SAG12*. This latter observation, in combination with the fact that the chlorophyll level was ~30% lower in severely N-stressed plants compared with plants grown under full N nutrition, indicates an induction of senescence-related parameters; however, as plants were chronically stressed for severe N limitation which caused a dramatic growth retardation (Bi *et al.*, 2007), a typical senescence induction programme that happens after leaves have undergone full expansion and maturation under well-fertilized conditions could not occur in this experiment.

In a further study, adult *Arabidopsis* plants were grown in hydroponic culture under a short-day life cycle in high-nitrate supply (6mM nitrate) for 5 weeks and subsequently starved on N-free medium for 10 d (Krapp *et al.*, 2011). Root and shoot samples were harvested after 1, 2, 4, and 10 d of starvation and analysed. The global transcriptomes of roots and shoots were determined at days 2 (mid-term) and 10 (long-term) of N starvation, and compared with the expression profiles before the onset of N starvation. Transcriptional responses were in general more rapid in roots, which were in direct contact with the culture medium, while global changes in the shoots were delayed. Of the 638 and 772 genes affected by N starvation in roots and leaves, respectively, only 142 were differentially expressed in both organs (of which 20 showed opposing responses in roots versus shoots). With respect to shoots, genes involved in ‘gluconeogenesis’, ‘sulphur assimilation’, and ‘minor carbohydrate metabolism’ were specifically over-represented after 2 d of N starvation, while genes involved in ‘fermentation’, ‘N metabolism’, ‘cofactor and vitamin metabolism’, ‘TCA cycle’, ‘transport’, and ‘RNA processing’ were significantly enriched after 10 d of starvation. These observations are consistent with the metabolic profiling results of both the current and previous studies (Watanabe *et al.*, 2013). The situation was, however, different for genes down-regulated during N starvation, where many genes affected after 2 d were also (but more prominently) changed after 10 d (Krapp *et al.*, 2011), including genes involved in ‘tetrapyrrole biosynthesis’, ‘photosynthesis’, ‘N metabolism’, ‘amino acid metabolism’, ‘cell wall’, and ‘lipid metabolism’. Furthermore, the functional classes ‘TCA cycle’, ‘glycolysis’, ‘redox’, ‘major carbohydrate metabolism’, ‘protein’, and ‘transport’ were specifically represented in the genes down-regulated in shoots after 10 d of N starvation. With respect to regulatory genes, only a relatively small number of genes was found among the most highly differentially expressed genes, including the MYB TF *PAP2*, an important regulator of anthocyanin biosynthesis genes, which was up-regulated in shoots. Few other TFs, including three CCAAT family members and *ECR1*, were up-regulated in roots, while *ANAC079*/*80* was down-regulated.

Krapp *et al.* (2011) also analysed the metabolic profiles in N-starved plants. In shoots, the total amino acid levels were not much affected during the first 24h of starvation, while thereafter levels declined rapidly, probably due to mobilization of leaf N resources to sustain root growth under these conditions. Similar to this report, a relatively rapid decline in total amino acid content in shoots of N-starved plants was observed in the present study. However, in contrast, in plants grown under continuous N limitation, higher total amino acid levels were found than in shoots of plants grown at high nitrate concentration, which was explained by a reduced utilization of amino acids for protein synthesis and growth (Tschoep *et al.*, 2009). Although total amino acids rapidly decreased upon N starvation in the present experiment, there were exceptions to this, including glutamate and glutamine, as well as tryptophan and tyrosine whose concentrations initially increased after N removal, but later on also decreased during extended N starvation. This differential response at the level of amino acids to N starvation is in accordance with the model that reduced growth under such conditions is due to changes in central metabolism rather than a general reduction in amino acids. Somewhat unexpected was the observation that the glutamate/glutamine ratio did not change in the experimental set-up, which is in contrast to previous findings where N limitation affected this ratio (Foyer *et al.*, 2003; Lemaitre *et al.*, 2008). Whilst this observation was somewhat unusual, it is important to note that all other markers of senescence displayed the expected changes, suggesting that this ratio may not be the most sensitive biomarker for senescence.

Besides a decline in total amino acids upon N removal from the medium, a reduction in the majority of the organic acids including pyruvate was also observed (Supplementary Table S9 at *JXB* online). The decline in pyruvate is in accordance with the strong reduction of alanine during N starvation. In contrast, the level of fructose strongly increased (~20-fold) in shoots during N limitation, and the concentration of several other sugars including sucrose, galactose, and to some extent glucose also increased upon N starvation. Additionally, the levels of GABA, threonate, ascorbate, and succinate were elevated. While these changes are striking, the exact mechanism underlying this phenomenon cannot be elucidated from the results in this study. There are, however, two possible explanations to account for the changes in the amounts of these metabolites. First, it is conceivable that the tricarboxylic acid (TCA) cycle is progressively down-regulated during the course of the extended N limitation, in an attempt to compensate for the increased availability of respiratory substrate. Secondly, the reduction of TCA cycle intermediates may be a consequence of a general down-regulation of biosynthetic metabolism which would be anticipated under conditions of N starvation. It is of interest that most of the free amino acids decreased significantly during N limitation, including arginine, asparagine, alanine, β-alanine, glycine, homoserine, proline, isoleucine, valine, phenylalanine, and valine, indicating either a reduced synthesis of amino acids or an increase of their degradation (Supplementary Table S9 available at *JXB* online).

An important result of this study is that the effect of N starvation on metabolism is rapidly reversed upon N resupply. Notably, the concentrations of almost all metabolites determined returned to values observed before the N limitation after 3 d of N resupply. Thus, from a metabolic standpoint, this time was enough for *Arabidopsis* to recover fully from the senescence that was clearly evident following N starvation.

The fact that *A. thaliana*, as well as crop species such as barley (Schildhauer *et al.*, 2008) or maize (Girardin *et al.*, 1985), and possibly other plants, has the ability to slow down an already initiated senescence programme or even reverse it when more environmentally benign conditions prevail (here: sufficient N supply) strongly indicates that having a regulatory system which allows the monitoring and integration of such environmental situations is of an evolutionary benefit. Further studies should focus on identifying and studying mutants that are impaired in the physiological response to N resupply. Currently, there are a number of mutants impaired in senescence (e.g. Guo and Gan, 2006; Kim *et al.*, 2009; Balazadeh *et al*., 2010, 2011; Zentgraf *et al.*, 2010; Lee *et al.*, 2012; Wu *et al.*, 2012); it would be highly interesting to test to what extent they contribute to reversion of senescence during N resupply.

## Supplementary data

Supplementary data are available at *JXB* online.

Figure S1. Expression levels of *SAG12* and *SEN1*.

Figure S2. Hormone contents.

Table S1. Genes differentially expressed upon limitation and resupply of nitrogen.

Table S2. Genes commonly up-regulated in the three senescence data sets.

Table S3. Genes commonly down-regulated in the three senescence data sets.

Table S4. Functions of genes specifically down- or up-regulated during N resupply.

Table S5. Gene expression profiles of the seven significant clusters 13, 16, 23, 25, 34, 44, and 47.

Table S6. Significantly over-represented gene ontology (GO) terms and enrichment of known *cis*-regulatory motifs.

Table S7. *De novo* motif prediction.

Table S8. Hormone contents.

Table S9. Primary metabolite profile of N deficiency-induced leaf senescence and its reversal by N resupply.

Table S10. Full primary metabolite data sets.

Supplementary Data
