# Supplementary material for: Reversal of senescence by N resupply to N-starved Arabidopsis thaliana: transcriptomic and metabolomic consequences
Source: J Exp Bot. 2014 Apr 1;65(14):3975–92. doi: 10.1093/jxb/eru119 (PMC4106441; doi:10.1093/jxb/eru119)
Supplement: Supplementary Data [file supp_65_14_3975__index.html]

Reversal of senescence by N resupply to N-starved Arabidopsis thaliana: transcriptomic and metabolomic consequences — Reversal of senescence by N resupply to N-starved Arabidopsis thaliana: transcriptomic and metabolomic consequences — Supplementary Data 

# Reversal of senescence by N resupply to N-starved *Arabidopsis thaliana*: transcriptomic and metabolomic consequences

## Supplementary Data

Data files

**Files in this Data Supplement:**

- Supplementary Data - Supplementary Data
- Supplementary Data - Supplementary Data
