# Supplementary material for: Reversal of senescence by N resupply to N-starved Arabidopsis thaliana: transcriptomic and metabolomic consequences
Source: J Exp Bot. 2014 Apr 1;65(14):3975–92. doi: 10.1093/jxb/eru119 (PMC4106441; doi:10.1093/jxb/eru119)
Supplement: Supplementary Data [file supp_eru119_jexbot117598_file002.pdf]

Supplemental Figure 1

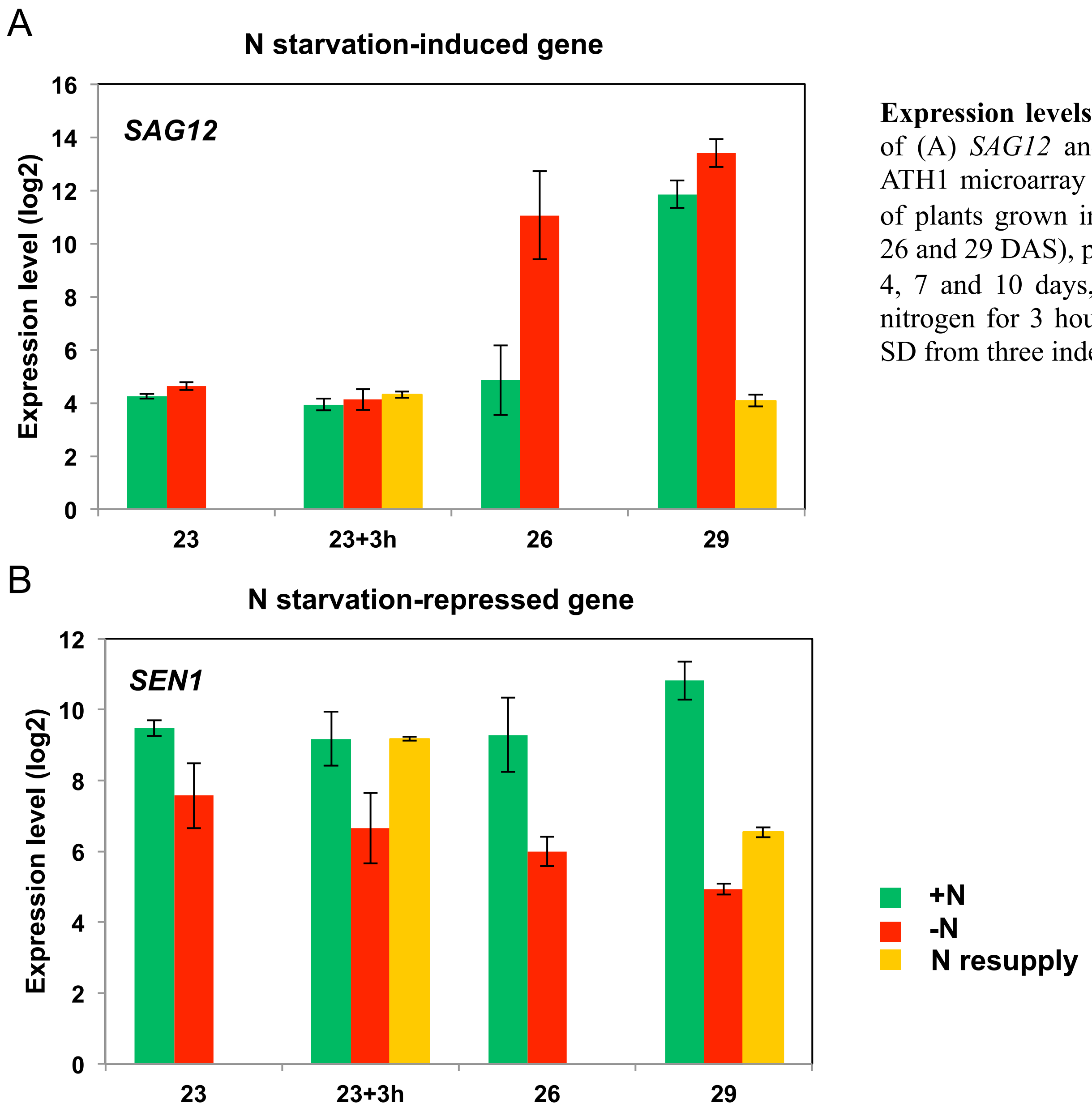

Expression levels of *SAG12* and *SEN1*. Transcript level of (A) *SAG12* and (B) *SEN1* determined by Affymetrix ATH1 microarray hybridisation in leaves number 1 and 2 of plants grown in full nitrogen medium (19, 23, 23+3h, 26 and 29 DAS), plants subjected to nitrogen starvation for 4, 7 and 10 days, and plants subjected to re-addition of nitrogen for 3 hours or 3 days. Values represent means ± SD from three independent sets of samples.

Supplemental Figure 2

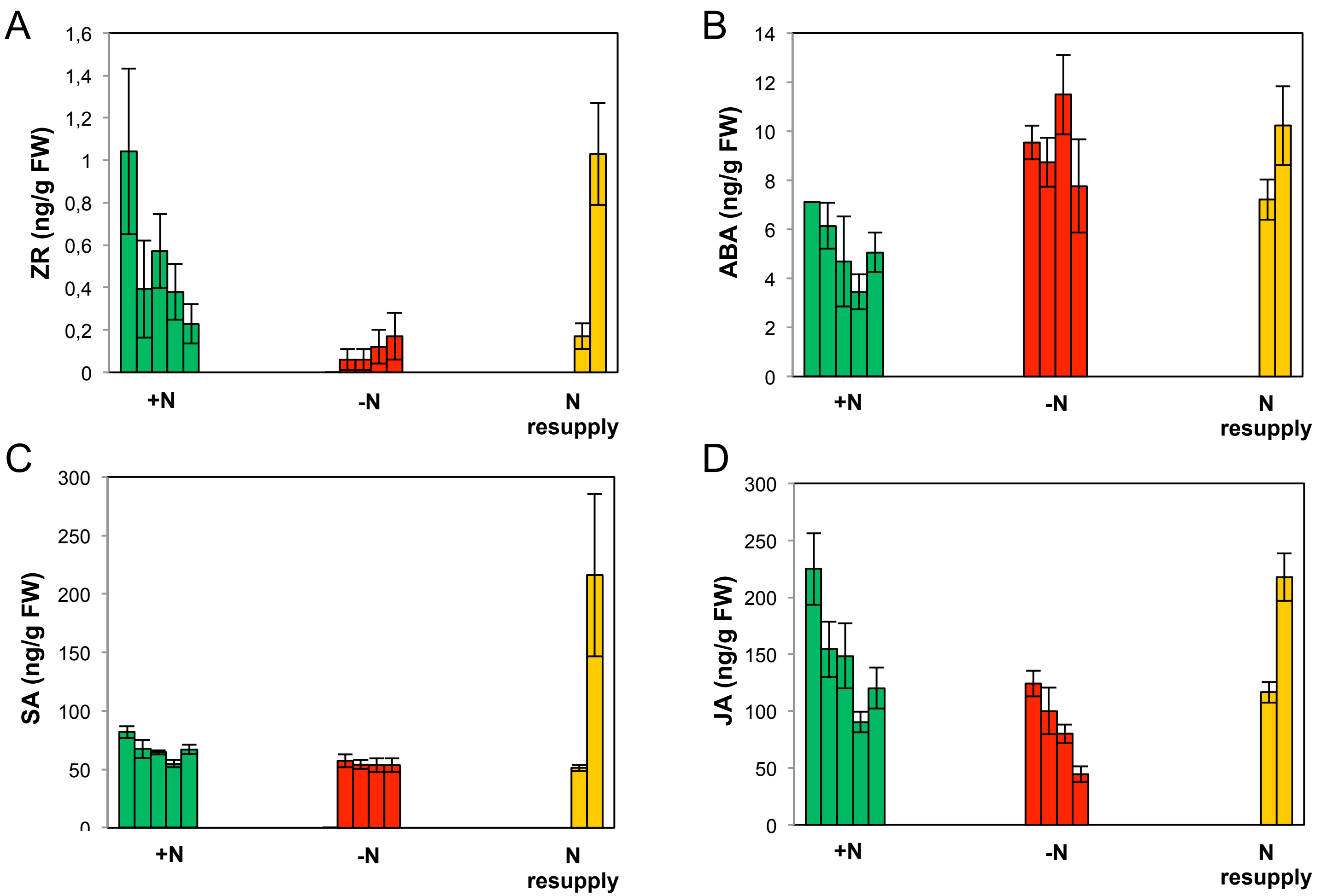

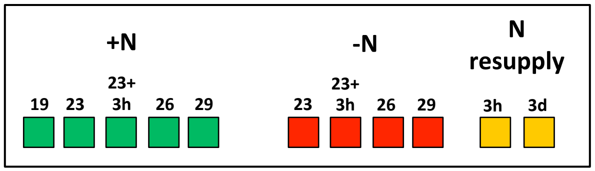

Hormone contents. Determination of (A) ZR, (B) ABA, (C) SA, and (D) JA in leaves number 1 and 2 of plants grown in full-nitrogen medium (19, 23, 26 and 29 DAS), plants subjected to nitrogen starvation for 4, 7 and 10 days, and those subjected to re-supply of nitrogen for 3 hours or 3 days. Values represent means ± SE from at least five independent sets of samples. FW, fresh weight.
